# Understanding cardiovascular aging as a disorder of mitochondrial network

**DOI:** 10.20517/jca.2026.07

**Published:** 2026-05-14

**Authors:** Taslima Akter Shila, Dhrubo Ahmad, Jianli Zhao, Huilan Tan, Bo Wang, John Zhang, Yajing Wang

**Affiliations:** Department of Biomedical Engineering, University of Alabama at Birmingham, Birmingham, AL 35294, USA.

**Keywords:** Cardiovascular aging, hypertension, mitophagy, endothelial dysfunction, mitochondrial dysfunction, epidemiology, heart failure with preserved ejection fraction

## Abstract

Cardiovascular aging is increasingly recognized as a mitochondrial-initiated systemic network dysfunction, a progressive, integrative failure driven by deteriorating mitochondrial quality and signaling. This review synthesizes emerging evidence linking comprehensive mitochondrial pathology to the erosion of cardiovascular resilience as a network-level dysfunction. Age-dependent remodeling of mitochondrial ultrastructure and component composition disrupts respiratory efficiency, positioning bioenergetic insufficiency as a central determinant of reduced stress tolerance across the cardiovascular system. Concurrently, defects in mitochondrial fission-fusion dynamics and impaired mitophagy propagate dysfunction within the mitochondrial network, amplifying the decline in energetic capacity. Beyond energy failure, the release of mitochondrial DNA, vesicles, and peptides activates innate immune sensors such as the cyclic guanosine monophosphate-adenosine monophosphate (GMP-AMP) synthase-stimulator of interferon genes (cGAS-STING) pathway, initiating chronic sterile inflammation that propagates maladaptive remodeling cascades throughout cardiovascular tissues and distal organs. We challenge the traditional view of mitochondria solely as energy producers, revealing that uncoupled perfusion and energy metabolism, together with nitric oxide imbalance, can serve as early indicators of diastolic dysfunction and ischemic susceptibility. Additionally, we introduce the concept of “mitochondrial age”, a composite measure that integrates respiratory function, imaging-based structural indices, and circulating mitochondrial biomarkers to quantify mitochondrial health. This metric may serve as a translational tool for assessing cardiovascular aging through mitochondrial network communication. Finally, we highlight rejuvenation strategies aimed at restoring mitochondrial youthfulness, ranging from behavioral interventions (exercise, time-restricted feeding) to metabolic and molecular therapies targeting nicotinamide adenine dinucleotide (NAD^+^) metabolism, mitophagy, and endothelial mitochondrial protection. Collectively, this review defines cardiovascular aging as a network-level mitochondrial disorder, offering new conceptual and therapeutic directions for preserving cardiac and vascular function.

## Graphical Abstract

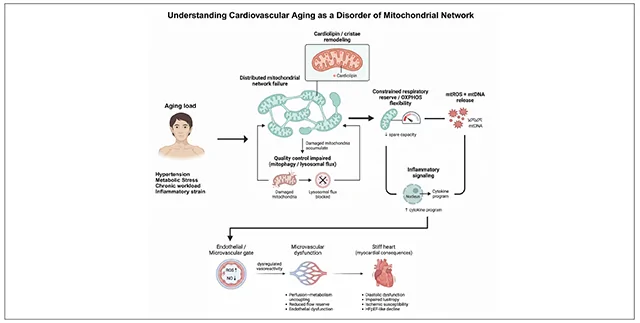

## INTRODUCTION

Aging remains the dominant risk factor for cardiovascular disease^[1,2]^, yet energetic biology is still too often treated as secondary to structural pathology. Here, we argue that cardiovascular aging is fundamentally a disorder of the mitochondrial network, characterized by declining bioenergetic reserve, impaired quality control, and maladaptive organelle-to-nucleus and organelle-to-immune signaling that progressively reduces cardiac and vascular resilience^[2–5]^. The implication is practical: delaying disease onset and preserving function will require preserving mitochondrial performance under stress, including efficient flux capacity, effective turnover, and controlled stress signaling.

The heart is uniquely susceptible to mitochondrial network disorders because mitochondria constitute a substantial proportion of cardiomyocyte volume and operate as an integrated cellular network that must synchronously sustain adenosine triphosphate (ATP) production, calcium handling, redox balance, and survival signaling on a beat-to-beat basis^[3,6,7]^. In aged rodent hearts and human myocardial samples, coordinated changes in cristae architecture and cardiolipin composition impair electron transport efficiency^[3,8]^ and energetic coupling^[8,9]^, while dysregulation of mitochondrial fission-fusion dynamics and insufficient mitophagy disrupt network quality control, allowing dysfunctional mitochondria to persist and propagate failure signals^[10,11]^. Importantly, these abnormalities extend beyond bioenergetics^[6,12]^. In murine models of cardiac injury and in cultured cardiomyocytes, damaged mitochondria release nucleic acids and peptides that activate innate immune pathways such as cyclic guanosine monophosphate-adenosine monophosphate (GMP-AMP) synthase-stimulator of interferon genes (cGAS-STING)^[13]^, linking mitochondrial dysfunction to chronic inflammation and impaired stress adaptation across cardiomyocytes, endothelial cells, and fibroblasts^[12,13]^. Together, these observations support the concept that cardiac aging and disease reflect not isolated mitochondrial defects, but a breakdown of mitochondrial-cellular network communication that progressively destabilizes tissue-level homeostasis.

An important conceptual distinction is whether mitochondrial dysfunction is a primary driver of cardiovascular aging or a downstream consequence of cumulative cellular damage. Current evidence supports a bidirectional model. In experimental systems, genetically or pharmacologically induced mitochondrial defects are sufficient to recapitulate key aging phenotypes, including reduced stress tolerance, impaired diastolic relaxation, endothelial dysfunction, and heightened inflammatory signaling, indicating that mitochondrial dysfunction can act as an initiating event^[14,15]^. Conversely, canonical aging processes such as telomere attrition, genomic instability, proteostatic decline, and chronic inflammatory stress progressively impair mitochondrial quality control, bioenergetics, and redox balance^[14,16,17]^. In this context, mitochondrial dysfunction becomes both a cause and an amplifier of aging phenotypes. The cardiovascular system is particularly susceptible to this feed-forward architecture because high energetic demand and continuous mechanical stress render mitochondria early sensors of strain and late executors of maladaptive remodeling. Thus, rather than framing mitochondrial decline strictly as cause or consequence, we interpret it as a rate-limiting node within a self-reinforcing aging network.

Clinically, the signature of this network failure is diffuse rather than focal: diastolic dysfunction precedes systolic loss^[18]^, microvascular rarefaction and endothelial stiffness impair perfusion, atrial substrate ages into fibrillation, and stress intolerance emerges years before overt heart failure. Across models and human studies, impaired bioenergetic reserve rather than resting ATP alone tracks most closely with functional decline. This shifts therapeutic priorities: the target is not “less reactive oxygen species (ROS)”, but more flexible, high-quality mitochondria capable of rapid flux, effective turnover, and calibrated retrograde signaling.

Promising avenues span behavior, pharmacology, and emerging biologics. Exercise and nutritional strategies can enhance mitochondrial biogenesis and turnover^[3,19,20]^. Agents that boost nicotinamide adenine dinucleotide (NAD^+^) or activate adenosine monophosphate -activated protein kinase (AMPK)/sirtuins (SIRTs) may restore the redox/acetylation balance^[21,22]^. Mitophagy inducers and cardiolipin-stabilizing approaches aim to improve organelle quality^[23]^. Endothelial-targeted interventions seek to rescue the microcirculation that governs myocardial oxygen delivery^[12]^. Yet translation has lagged because trials seldom measure mitochondrial function directly, interventions often ignore cell-type and subcellular heterogeneity (intermyofibrillar *vs.* subsarcolemmal pools; cardiomyocyte *vs.* endothelial mitochondria), and endpoints capture downstream pathology rather than mitochondrial youthfulness.

This review synthesizes a network-centric view of cardiovascular aging and organizes the field around three axes: energy, quality control, and signaling to unify disparate observations. We propose practical metrics to quantify “mitochondrial age”, highlight crosstalk between cardiovascular mitochondria and remote organs as an early and tractable target, and outline a framework for cellular mitochondrial crosstalk and its translational implications that prioritizes function over surrogate biomarkers. Making the heart’s mitochondria functionally younger, rather than merely slowing the organismal aging, should become a central objective of cardiovascular aging research and therapy.

## KEY RECOGNITIONS IN MITOCHONDRIAL NETWORKS AND CARDIAC AGING

### Mitochondrial networks as an organizing principle

An important recognition emerging from the past decade is that mitochondrial dysfunction in the aging heart is not well captured by single readouts of “mitochondrial content”, resting ATP levels, or bulk oxidative stress, but instead reflects failure modes of a spatially organized mitochondrial network^[24,25]^. Cardiac mitochondria operate as an interconnected and dynamically reconfiguring system that must match energy production to rapidly fluctuating demands while simultaneously coordinating calcium handling, redox buffering, and stress signaling. Aging perturbs these network properties through cumulative alterations in ultrastructure, membrane composition, and protein homeostasis^[26]^ with downstream consequences that depend on network topology and connectivity rather than on the mean function alone^[24,27,28]^. This perspective provides a mechanistic basis for why bioenergetic reserve and stress responsiveness can decline despite apparently preserved baseline energetics, and it reframes mitochondrial pathology as a systems-level determinant of reduced myocardial resilience rather than as an epiphenomenon of end-stage disease.

### Heterogeneity, crosstalk, and network failure across cardiac cell types

A second key recognition is that mitochondrial network dysfunction in cardiac aging is intrinsically heterogeneous, both among mitochondrial subpopulations and within the cellular myocardial ecosystem^[3,29]^. Intermyofibrillar and subsarcolemmal mitochondrial pools exhibit distinct biophysical constraints, substrate access, and turnover dynamics, and these differences likely shape how energetic insufficiency and quality-control failure manifest during aging^[30,31]^. Moreover, cardiomyocyte mitochondrial decline interacts with, and may be initiated or amplified by, parallel dysfunction in endothelial and microvascular mitochondria that govern perfusion-metabolism coupling and nitric oxide (NO) signaling, thereby influencing early diastolic impairment and ischemic susceptibility^[12,32,33]^. Aging-associated mitochondrial stress also propagates through retrograde communication pathways, including mitochondrial-derived ligands that activate innate immune sensing and remodel transcriptional programs, linking organelle dysfunction to inflammaging, fibrosis, and maladaptive remodeling^[13,34]^. Together, these insights position the mitochondrial network distributed across cell types and integrated through metabolic and inflammatory crosstalk as a central explanatory framework for cardiac aging and a rational substrate for interventions aimed at preserving function.

In addition to direct cellular interactions, recent studies have recognized the role of mitochondrial transfer and extracellular vesicles (EVs) as significant mechanisms of intercellular mitochondrial communication in cardiac aging^[35–37]^. Intercellular mitochondrial transfer refers to the process through which functional mitochondria or mitochondrial components are transferred from donor cells to recipient cells, allowing for mitochondrial rescue or reprogramming. This form of mitochondrial transplantation occurs through direct cell-to-cell connections (such as gap junctions) or via vesicular transfer, with emerging evidence suggesting that damaged or stressed cells can export mitochondria to healthy cells to mitigate dysfunction^[38,39]^. Mitochondria are also transported in EVs, including both small EVs (such as exosomes) and larger vesicles, known as exospheres, which are capable of packaging entire mitochondria or mitochondrial components^[35,40,41]^. These vesicles serve as carriers of mitochondrial material and signaling molecules, facilitating the transfer of mitochondria between cells and enabling the adaptation of recipient cells to metabolic stress^[42,43]^. This process not only promotes mitochondrial rejuvenation but also helps coordinate cellular responses to stress, inflammation, and metabolic demands^[36,37,43]^. Recent findings suggest that the transfer of mitochondria via EVs can influence cardiomyocyte function, endothelial cell behavior, and even the microvascular environment, providing a novel layer of complexity in the understanding of intercellular communication and network dysfunction in cardiac aging^[39,43]^. The ability of mitochondria to be transferred via these mechanisms represents a form of mitochondrial network maintenance, and dysfunction in this process may contribute to the maladaptive remodeling observed in aging and heart disease.

## CURRENT VIEW: CARDIOVASCULAR AGING AND MITOCHONDRIAL IMBALANCE

**Figure 1.**
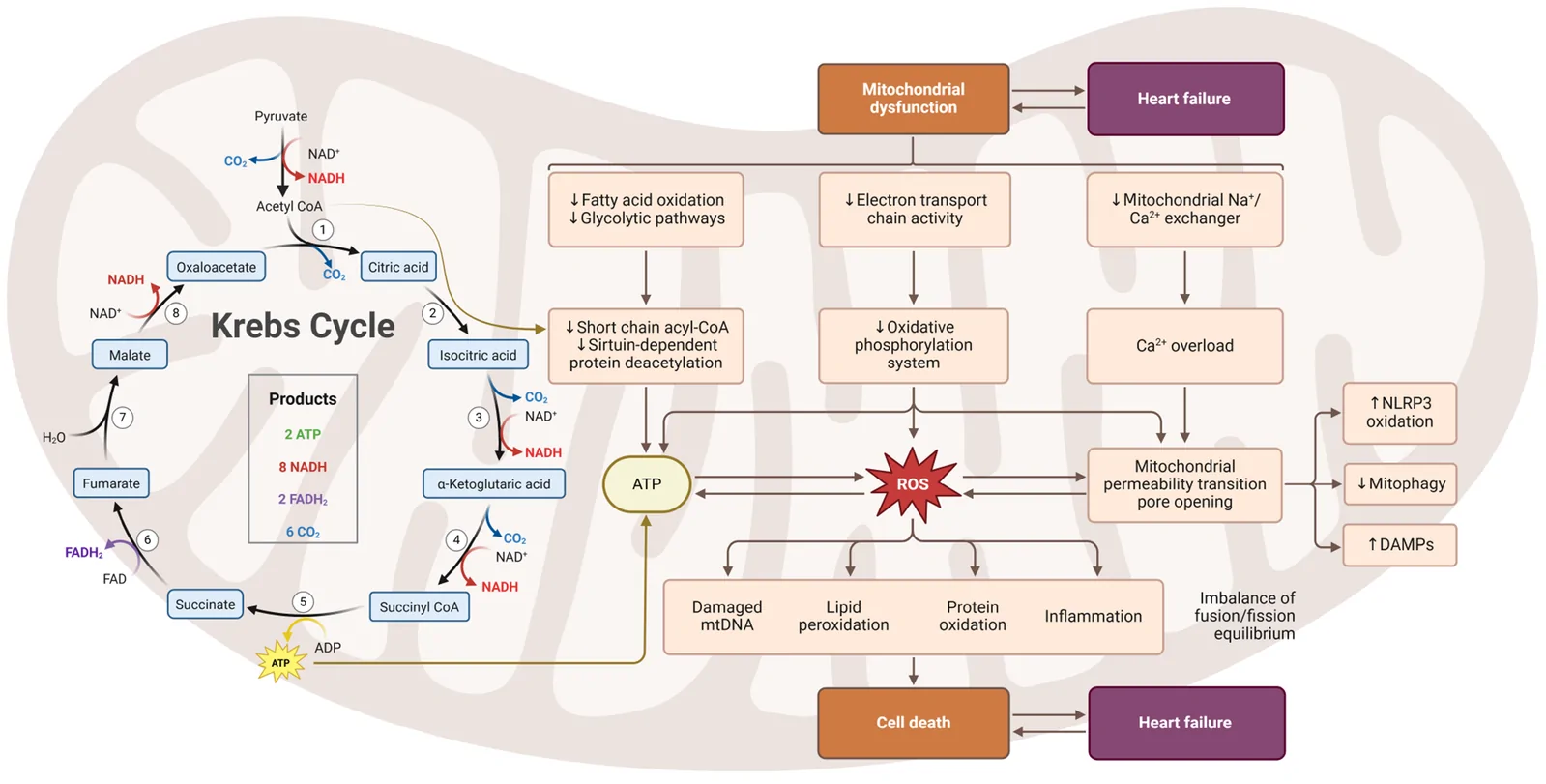
Mitochondrial metabolic dysfunction and redox-driven injury pathways in cardiovascular aging resulting in heart failure. (Created in BioRender. Ahmad D (2026) https://BioRender.com/61c7ow3). NADH: Nicotinamide adenine dinucleotide; FAD: flavin adenine dinucleotide; ATP: adenosine triphosphate; mtDNA: mitochondrial DNA; DAMPs: danger-associated molecular patterns; ROS: reactive oxygen species; NLRP3: nucleotide-binding oligomerization domain-like receptor family pyrin domain-containing 3

A prevailing framework now interprets cardiovascular aging as a progressive mitochondrial imbalance in which the capacity to generate ATP adaptively, maintain organelle integrity, and constrain stress-evoked signaling becomes insufficient to meet the cumulative workload and damage^[44]^. This view is supported by the observation that aging phenotypes are expressed most clearly as deficits in functional reserve, exercise intolerance, reduced ischemic tolerance, and endothelial dysfunction rather than as isolated reductions in resting ATP^[45]^. Mechanistically, mitochondrial imbalance is increasingly defined by three interlocking features: (i) constrained oxidative phosphorylation response to demand^[3]^, (ii) reduced renewal throughput (dynamics and mitophagy)^[46]^, and (iii) amplified retrograde signaling that biases tissues toward sterile inflammation and remodeling^[5,34,47]^ [Figure 1]. Importantly, this model accommodates the multi-organelle nature of aging while proposing that mitochondria function as a rate-limiting integrator of energetic, redox, and inflammatory cues across cardiomyocytes and the vascular wall.

### Energetic constraint: inner membrane remodeling and metabolic control

In aged rodent myocardium and ex vivo human cardiac tissue studies, age-related energetic decline is increasingly attributed to mechanisms that restrict peak respiratory flux and coupling efficiency^[3]^. Structural remodeling of the inner mitochondrial membrane (IMM), including disrupted cristae architecture and altered membrane composition, can impair electron transport organization and elevate electron leak under stress^[48]^. Cardiolipin is a key molecular node because it stabilizes multiple respiratory complexes and supports inner-membrane curvature; remodeling or oxidation of cardiolipin can therefore reduce oxidative phosphorylation efficiency and contribute to decreased stress tolerance^[49]^. In parallel, aging perturbs metabolic control systems that tune mitochondrial enzymes and redox state^[50]^. Declining NAD^+^ availability and altered downstream signaling [SIRT-dependent deacetylation programs, adenosine monophosphate-activated protein kinase (AMPK)-linked nutrient sensing] alter mitochondrial protein acetylation, substrate oxidation, and antioxidant defense capacity, thereby narrowing the dynamic range of mitochondrial output^[50,51]^. The net effect is a disproportionate loss of reserve capacity that is most evident during physiological challenge rather than at baseline.

### Turnover bottlenecks: dynamics, mitophagy, and lysosomal throughput

Mitochondrial quality control depends on coordinated processes that preserve organelle structure, segregate damaged components, and remove dysfunctional mitochondria. Mitochondrial dynamics refers to the continuous cycles of fission and fusion that remodel the mitochondrial network, allowing content mixing, dilution of damage, and isolation of dysfunctional segments. These processes are governed primarily by large GTPases, including dynamin-related protein 1 (DRP1) (fission), mitofusin 1/2 (MFN1/2), and optic atrophy 1 (OPA1) (fusion), and are essential for maintaining bioenergetic efficiency and structural integrity in highly energy-demanding tissues such as the heart^[52–54]^. Mitophagy complements these processes by selectively removing damaged or bioenergetically compromised mitochondria through phosphatase and tensin homolog (PTEN)-induced putative kinase 1 (PINK1)/Parkin-dependent ubiquitin signaling or receptor-mediated pathways such as BCL2 interacting protein 3 (BNIP3)/NIP3-like protein X (NIX) and FUN14 domain-containing 1 (FUNDC1). Together, mitochondrial dynamics and mitophagy form an integrated quality-control system that preserves respiratory reserve, limits excessive ROS production, and restrains inflammatory signaling. Disruption of either arm has been strongly implicated in cardiovascular aging and disease^[54–57]^.

A second core association is the reduction in effective mitochondrial renewal relative to damage burden. Mitochondrial fission-fusion machinery, including DRP1, MFN1/2, and OPA1, maintains network function by enabling content complementation, segregation of damaged segments, and coordination with biogenesis and autophagic clearance^[58]^. Aging frequently perturbs this coupling, promoting fragmentation and impairing functional complementation, while mitophagy flux becomes insufficient to remove dysfunctional or damaged organelles^[46,59]^. This insufficiency can arise at several points, including mitochondrial damage sensing and ubiquitin signaling (PINK1/Parkin)^[60]^, receptor-mediated pathways such as BNIP3/NIX, FUNDC1^[61]^, or downstream autophagosome-lysosome clearance, yet it converges on persistence of dysfunctional mitochondria that depress respiratory reserve and amplify redox stress^[62,63]^. This perspective is particularly important in aging because it suggests that interventions must restore quality-control flux rather than merely increase mitochondrial content.

### Integration of mitochondrial quality control systems

Mitochondrial quality control in the aging cardiovascular system is best understood not as a collection of independent pathways but as an integrated network of interdependent regulatory modules. Core components of this network include mitochondrial dynamics (the balance between fission and fusion), mitophagy, metabolic signaling pathways such as NAD^+^-dependent SIRT signaling and AMPK signaling, and lysosomal clearance capacity. These systems are tightly coupled such that dysfunction in one node can propagate failure across the network^[52]^. For example, declining NAD^+^ availability with aging impairs the activity of SIRT deacetylases, particularly SIRT1 and SIRT3, which regulate transcriptional programs controlling mitochondrial biogenesis and autophagic turnover. Reduced SIRT signaling therefore diminishes the transcriptional and post-translational regulation of mitophagy components, including PINK1/Parkin and receptor-mediated pathways such as BNIP3 and FUNDC1^[51,64]^. In parallel, energetic stress and impaired AMPK activation can reduce mitochondrial fission events necessary for isolating damaged mitochondrial segments prior to autophagic removal^[65,66]^. When these regulatory layers fail simultaneously, dysfunctional mitochondria accumulate, producing excess ROS and releasing mitochondrial danger signals that further amplify inflammatory and metabolic stress. This interdependence suggests that mitochondrial aging is not simply an additive decline of individual pathways but rather a systems-level collapse of quality-control coordination. Consequently, therapeutic strategies that restore upstream regulators of mitochondrial maintenance, such as NAD^+^ metabolism or AMPK signaling, may have disproportionately broad effects by simultaneously improving mitochondrial turnover, metabolic flexibility, and inflammatory control [Table 1].

### Mitochondrial communication networks and vascular gating

Mitochondrial communication in cardiovascular aging is enabled by the organelle’s compartmental design and by specialized interfaces that couple bioenergetic state to cytosolic, nuclear, and extracellular programs. Structurally, the outer mitochondrial membrane (OMM) defines a signaling and trafficking boundary enriched in permeability and docking proteins, the intermembrane space (IMS) functions as a redox-sensitive relay compartment, and the IMM is folded into cristae that spatially organize electron transport, proton pumping, and ATP synthase into high-efficiency microdomains^[67]^. The matrix contains the tricarboxylic acid (TCA) cycle, pathways for handling fatty-acid-derived acetyl units, one-carbon metabolism modules, and the mitochondrial genome packaged into nucleoids^[68]^. This architecture matters because most “messages” originate as compartment-specific deviations in membrane potential, reduced nicotinamide adenine dinucleotide (NADH)/NAD^+^ balance, ROS tone, calcium (Ca^2+^) flux, and metabolite pools. These changes are generated locally and are exported or transduced across membranes through defined conduits, rather than by diffuse equilibration^[68]^.

**Figure 2.**
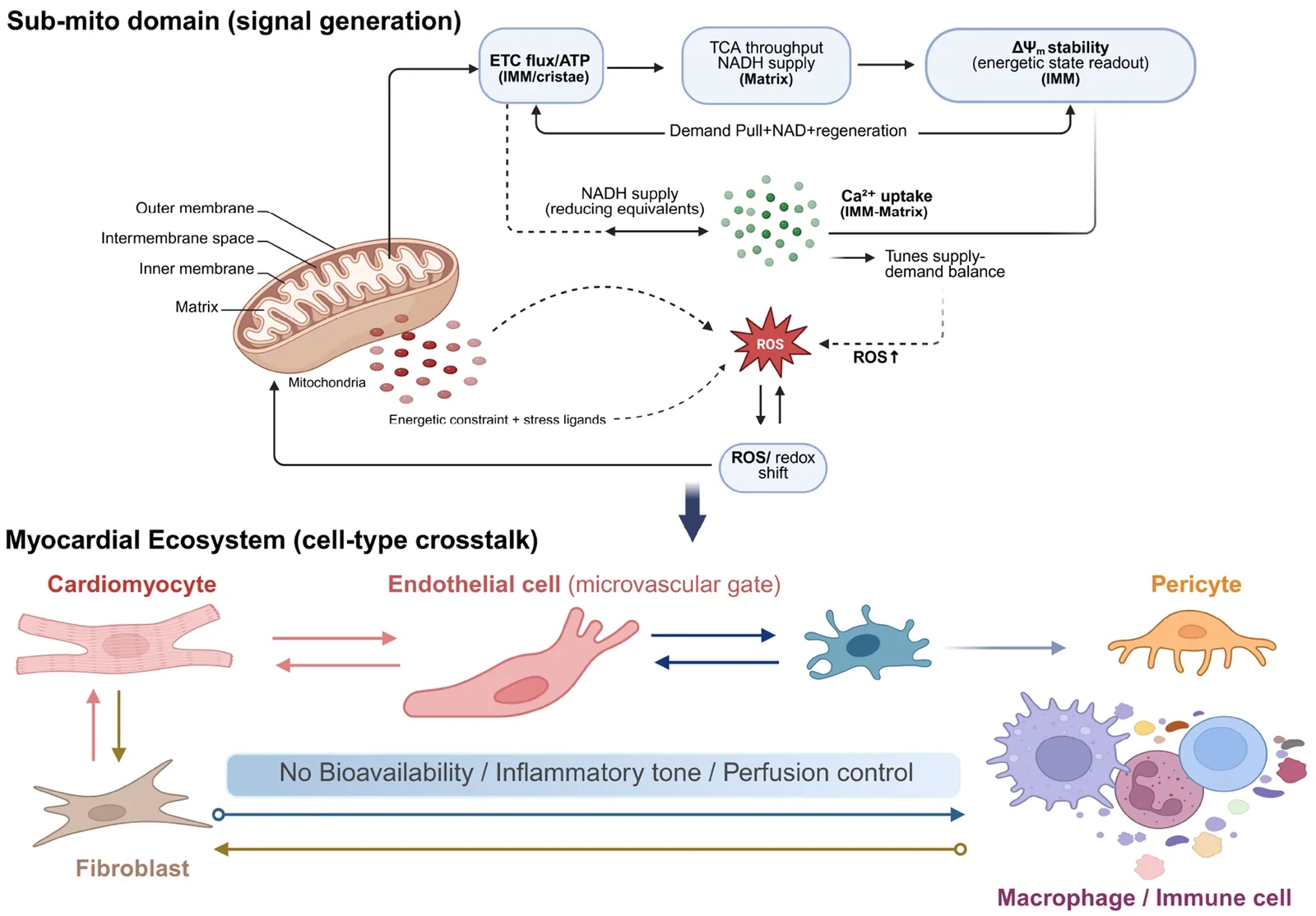
Sub-mitochondrial signal generation and myocardial cell-type crosstalk in cardiovascular aging. (Created in BioRender. Shila TA (2026) https://BioRender.com/2bztp85). ETC: Electron transport chain; IMM: inner mitochondrial membrane; ATP: adenosine triphosphate; TCA: tricarboxylic acid; NADH: nicotinamide adenine dinucleotide; ROS: reactive oxygen species.

At the sub-mitochondrial level, metabolic crosstalk is dominated by a matrix-to-IMM coupling loop in which energetic demand, redox tone, and Ca^2+^ handling are integrated across sub-mitochondrial domains [Figure 2]. In this loop, matrix dehydrogenase activity sets reducing-equivalent supply, while IMM respiratory flux sets demand and leak^[69]^. Ca^2+^ uptake is a key control knob for this loop because mitochondrial Ca^2+^ entry tunes Ca^2+^-sensitive matrix dehydrogenases and thereby adjusts NADH regeneration during stress^[70]^, while dysregulated Ca^2+^ handling increases ROS production and destabilizes energetic control^[70]^, shifting mitochondria from adaptive signaling to damage amplification. The relevance for vascular aging is that endothelial mitochondria often operate near signaling thresholds, where modest shifts in redox or Ca^2+^ microdomains can reprogram NO bioavailability, inflammatory tone, and barrier behavior^[12]^.

High-bandwidth organelle-to-organelle crosstalk is then implemented through physical contact sites rather than through bulk diffusion^[71]^ [Figure 2]. The best-characterized interface is the endoplasmic reticulum (ER)-mitochondria contact domain, often termed the mitochondria-associated membrane (MAM)^[71]^, where ER Ca^2+^ release channels are functionally coupled to OMM uptake routes^[72]^. A canonical example is the inositol 1,4,5-trisphosphate receptor (IP3R)-glucose-regulated protein 75 (GRP75)-voltage-dependent anion channel 1 (VDAC1) axis, which positions ER Ca^2+^ release near mitochondrial entry pathways so that mitochondria can sample high local Ca^2+^ microdomains despite the low affinity of downstream uptake machinery^[72]^. This arrangement supports physiological metabolic matching, but it also creates a direct route through which chronic ER stress or dysregulated Ca^2+^ release can drive mitochondrial Ca^2+^ overload, ROS escalation, and downstream inflammatory signaling^[72]^.

Quality control and cargo routing provide a second inter-organelle communication layer that is essential for interpreting which mitochondrial components are presented to the cell. When damage is focal or sublethal, mitochondria can generate mitochondria-derived vesicles (MDVs) that selectively package oxidized proteins or other cargo for trafficking to endolysosomal compartments^[73]^. This process provides a rapid, graded disposal pathway that can precede or complement whole-organelle mitophagy. Importantly, MDV biology also links mitochondrial quality control to immune signaling and to EV composition, indicating that mitochondrial communication includes selective export decisions, not only internal degradation choices^[74]^.

Retrograde signaling to the nucleus can be conceptualized as three partially separable output classes. The first is metabolite-to-epigenome coupling. Mitochondrial metabolism supplies or regulates cofactors and intermediates that directly constrain chromatin-modifying enzymes and transcriptional competence, including acetyl-Co-enzyme A, α-ketoglutarate, succinate, fumarate, and NAD^+^-linked pathways. In practical terms, mitochondrial flux states can bias the capacity for histone acetylation and demethylation, thereby shifting transcriptional set points even in the absence of overt cell death or inflammation^[75]^. This coupling provides a mechanistic bridge from mitochondrial substrate handling to durable vascular and myocardial remodeling programs. The second is stress transduction via dedicated relay pathways, exemplified by DAP3 binding cell death enhancer 1 (DELE1)-dependent activation of the heme-regulated inhibitor (HRI) branch of the integrated stress response during mitochondrial import or proteostasis perturbations^[76]^. This pathway converts mitochondrial distress into coordinated translational and transcriptional remodeling, including endocrine-like outputs, and helps explain how chronic, sublethal mitochondrial stress can become a stable cellular phenotype^[76]^. The third output class is innate immune activation when compartmental containment fails. In murine models of pressure overload, myocardial infarction, and in vitro cardiomyocyte injury, the release of oxidized mitochondrial DNA (mtDNA) into the cytosol can activate cGAS-STING signaling and downstream interferon and nuclear factor kappa B (NF-κB) programs, linking mitochondrial injury to sterile inflammation, fibroblast activation, and extracellular matrix remodeling in cardiovascular disease settings^[77]^.

These mechanistic layers also clarify why intact mitochondria generally do not circulate in blood as stable, freely functional units. Outside the cellular environment, mitochondria are deprived of the ionic buffering, substrate provisioning, and chaperone-supported membrane maintenance required for sustained oxidative phosphorylation^[78]^. In addition, mitochondrial surfaces and contents are immunologically conspicuous because mitochondrial molecules share features with bacterial motifs, making extracellular mitochondria prone to being interpreted as danger signals and rapidly cleared^[79]^. Consistent with this, bioenergetic characterization of circulating cell-free mitochondria in human blood argues against robust respiratory competence *in vivo*, supporting a model in which most extracellular mitochondrial material reflects release and processing rather than the purposeful circulation of fully functional organelles.

**Figure 3.**
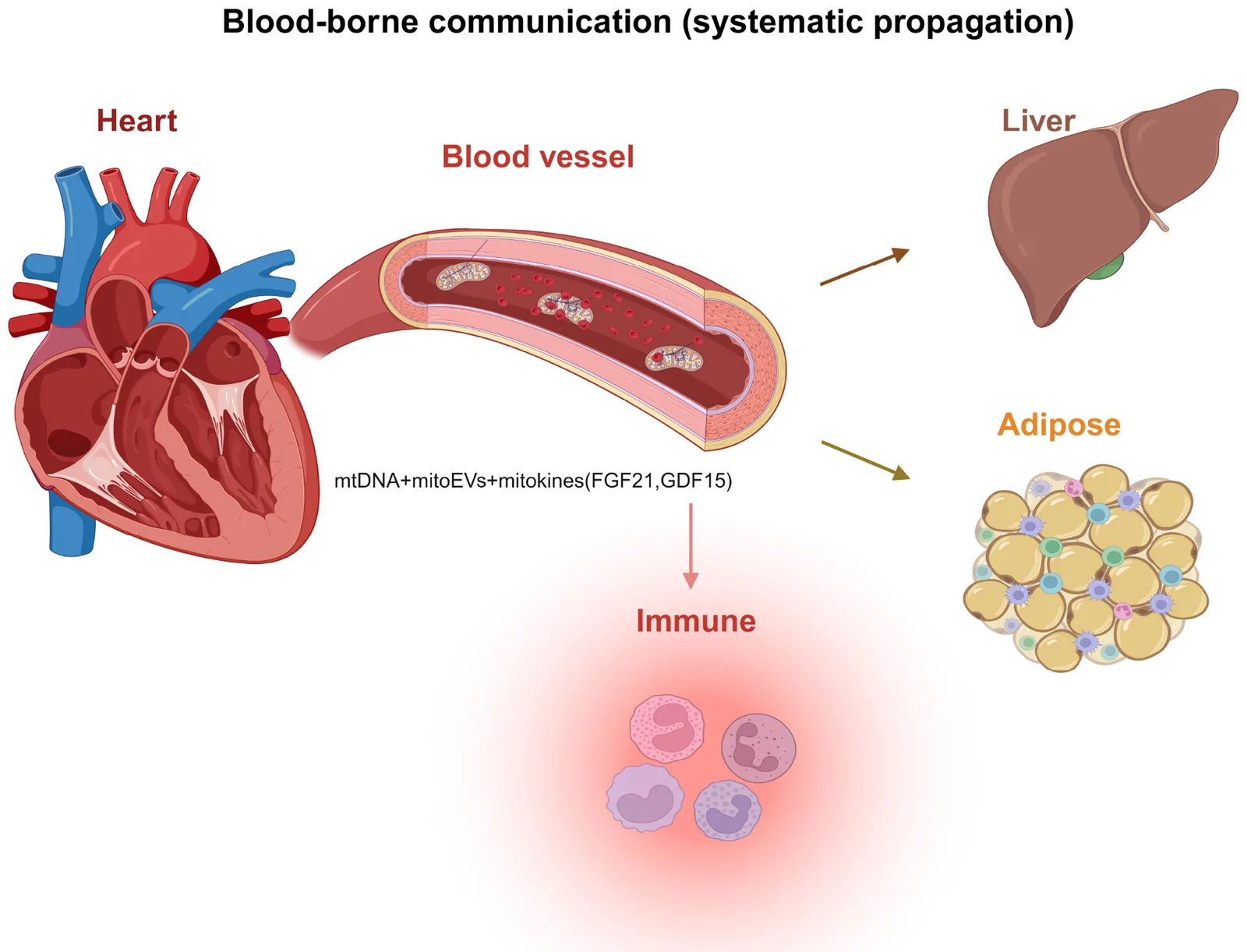
Blood-borne propagation of mitochondrial stress signals from the heart to peripheral organs. (Created in BioRender. Shila TA (2026) https://BioRender.com/94twtl3). mitoEVs: Mitochondrial extracellular vesicles; mtDNA: mitochondrial DNA; FGF21: fibroblast growth factor 21; GDF15: growth differentiation factor 15.

What circulates effectively are mitochondrial signals in forms that are smaller, stabilized, or packaged^[36]^. These include mtDNA fragments, mitochondrial EVs, and stress-induced mitokines that propagate cardiac mitochondrial stress to distal organs [Figure 3]. One major class is cell-free mitochondrial nucleic acids, particularly mtDNA fragments, which can act as inflammatory ligands and have emerged as biomarkers of tissue stress and immune activation in blood^[79]^. The second class is vesicle-associated mitochondrial cargo. Reviews describe mitochondrial extracellular vesicles (mitoEVs) and related vesicular populations as mediators of immune responses and bioenergetic remodeling, with growing emphasis on their potential as aging biomarkers^[36]^. Mechanistic work further supports that selective packaging of mitochondrial proteins into EVs can depend on MDV pathways, implying that mitochondrial material can be exported in a regulated, pathway-specific manner rather than by nonspecific rupture alone^[74]^. A third class comprises endocrine-like “mitokines” induced by mitochondrial stress programs, most prominently fibroblast growth factor 21 (FGF21) and growth differentiation factor 15 (GDF15), which are increasingly positioned as systemic readouts of mitochondrial stress and as mediators of organism-level metabolic adaptation but may become maladaptive when chronically elevated^[80]^.

In this framework, the mitochondrial network communicates with multiple partners across scales, and vascular gating arises because endothelial and microvascular cells sit at a convergence point for metabolic sensing, inflammatory routing, and flow control. Intracellularly, endothelial mitochondria integrate ER-derived Ca^2+^ microdomains at MAMs with redox control and quality-control routing, shaping NO biology and inflammatory responsiveness. Tissue-level propagation occurs through paracrine cytokines, vesicle traffic, and immune recruitment^[81]^ but also through direct microvascular cell-to-cell conduits that can transmit metabolic influence. For example, pericyte-to^[82]^-endothelial communication via tunneling nanotubes has been described with measurable effects on endothelial metabolism^[83]^, emphasizing that microvascular energetic phenotypes can be coordinated through physical intercellular structures rather than through soluble mediators alone. Systemically, blood-borne mtDNA, mitoEV cargo, and mitokines couple local mitochondrial stress to distal immune and metabolic responses, creating feedback loops that can stabilize sterile inflammation and impair perfusion-energy matching when mitochondrial injury becomes persistent.

### Mitochondria as central drivers of cardiovascular aging

A useful conceptual distinction is that mitochondrial signaling during aging evolves from an initially adaptive stress-response program to a maladaptive amplifier of tissue dysfunction. In the early stages of cardiovascular aging, moderate mitochondrial stress can activate protective signaling pathways, including transient increases in ROS that stimulate antioxidant defenses, enhanced mitochondrial turnover through mitophagy, and induction of mitochondrial stress responses such as mitokine signaling (e.g., FGF21 and GDF15). These responses can temporarily preserve cellular homeostasis by promoting metabolic flexibility and the removal of damaged organelles^[84,85]^. However, as aging progresses and mitochondrial damage accumulates, these same pathways become chronically activated. Persistent mtDNA release, sustained cGAS-STING signaling, and impaired mitophagy shift mitochondrial signaling toward a maladaptive state characterized by sterile inflammation, endothelial dysfunction, and reduced bioenergetic reserve. In this later stage, mitochondrial signaling no longer supports adaptation but instead reinforces fibrotic remodeling, microvascular dysfunction, and impaired cardiac stress tolerance^[5,86]^.

Mitochondrial network dysfunction also interacts closely with traditional cardiovascular risk factors, particularly hypertension and diabetes. Chronic pressure overload in hypertension increases myocardial energetic demand while simultaneously promoting mitochondrial oxidative stress and endothelial dysfunction, which together impair NO signaling and vascular relaxation^[87,88]^. Similarly, in diabetes and insulin resistance, mitochondrial substrate overload and altered fatty acid oxidation drive excessive electron transport chain (ETC) flux and ROS generation, contributing to mtDNA damage and impaired quality-control pathways. These cardiometabolic conditions, therefore, accelerate mitochondrial aging by compounding defects in redox regulation, NAD^+^ signaling, and mitochondrial turnover, ultimately reducing the capacity of the mitochondrial network to maintain perfusion-energy coupling under physiological stress^[89]^.

A growing body of evidence places mitochondria at the center of cardiovascular aging. In the heart and vasculature, aging is accompanied by declining respiratory efficiency^[3]^, remodeling of cristae and cardiolipin^[3]^, imbalance in fission-fusion dynamics, and shortfalls in mitophagy^[46]^. These changes erode bioenergetic reserve and blunt stress-adaptative responses, which are more accurately reflected by dynamic mitochondrial capacity than by resting ATP levels alone. Recent syntheses frame these alterations as a coordinated network rather than isolated defects^[56]^, shifting the field’s focus beyond ROS toward mitochondrial quality control and flexibility. Vascular aging provides an early, clinically salient readout of this biology. Large-artery stiffening and endothelial dysfunction emerge through intertwined pathways linking inflammation, redox imbalance, and dysregulated energy-sensing nodes (e.g., AMPK and SIRTs). At the microvascular level, rarefaction and endothelial senescence impair perfusion-metabolism matching and are increasingly recognized in phenotypes such as heart failure with preserved ejection fraction (HFpEF).

Mitochondria also act as immunometabolic signaling hubs. Leakage of mtDNA and other danger signals can activate cGAS-STING and related pathways, sustaining the sterile inflammation typical of aging tissues, including heart and vessels^[5]^. This signaling axis is currently the main focus of therapeutic exploration in cardiovascular disease. The current review synthesizes evidence across epidemiology, clinical research, and experimental cardiology to (i) frame cardiovascular aging as a disorder of an integrated mitochondrial vascular network, (ii) distill advances in mitochondrial quality control, bioenergetic reserve, and innate immune signaling as they pertain to cardiac and vascular aging, and (iii) outline implications for prevention and therapy in older adults, with particular attention to strategies that enhance mitochondrial flexibility and microvascular function. By integrating these strands, this review seeks to identify practical opportunities for measurement and intervention that can improve cardiovascular health span.

### Therapeutic opportunities for mitochondrial aging in cardiovascular disease

Therapeutic strategies for mitochondrial aging in cardiovascular disease are shifting from broad, nonspecific “mitochondrial support” toward interventions that target defined mitochondrial pathways and can be evaluated with measurable human endpoints. In vascular aging, mitochondria-targeted redox modulation has advanced beyond preclinical rationale: chronic supplementation with the mitochondrial antioxidant mitoquinone (MitoQ) improved brachial artery flow-mediated dilation and reduced aortic stiffness in healthy late middle-aged and older adults in a randomized controlled study, and this line of work has expanded into protocols designed to test vascular function outcomes in older cohorts.

In parallel, quality-control therapeutics that enhance mitochondrial turnover have entered human testing. Urolithin A, a mitophagy-linked intervention, improved muscle endurance and shifted circulating biomarkers consistent with enhanced mitochondrial health in randomized trials in older adults, offering proof-of-concept that organelle quality control can be modulated safely with functional readouts in humans^[90]^. NAD^+^-repletion strategies similarly demonstrate reliable target engagement in humans, and recent translational efforts increasingly evaluate blood pressure and arterial stiffness as mechanistically relevant vascular surrogates; a pilot randomized clinical trial combining nicotinamide riboside (NR) with supervised exercise in middle-aged and older adults with hypertension exemplifies this mechanistic, endpoint-linked approach^[91]^.

Dietary timing interventions are also being tested with mechanistic vascular endpoints in older populations. Notably, months-long time-restricted eating protocols have been designed specifically to examine whether sustained adherence improves endothelial function and neurovascular or cerebrovascular coupling in community-dwelling older adults^[92]^.

**Figure 4.**
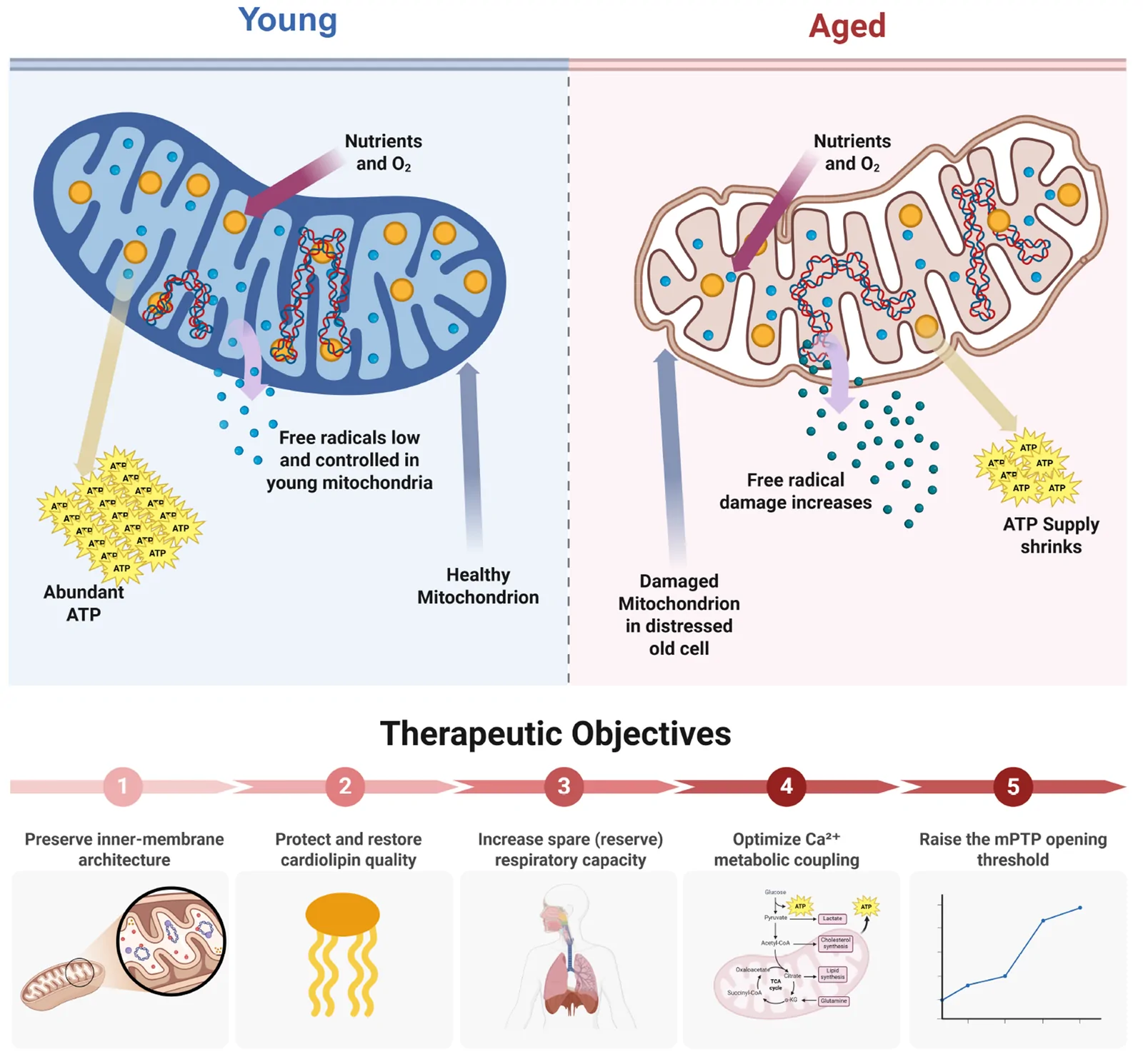
Structural and functional remodeling of mitochondria with aging and corresponding therapeutic objectives. (Created in BioRender. Ahmad D (2026) https://BioRender.com/p7qla0o). ATP: Adenosine triphosphate; TCA: tricarboxylic acid; mPTP: mitochondrial permeability transition pore; α-KG:

At the pharmacologic end of the spectrum, inner-membrane and cristae-directed stabilization reached a major regulatory milestone in 2025, when the U.S. Food and Drug Administration granted accelerated approval to Forzinity (elamipretide) injection for Barth syndrome, highlighting the clinical tractability of therapies that directly target mitochondrial inner-membrane structure and function^[93]^. Finally, established cardiometabolic agents are increasingly interpreted through mitochondrial and endothelial mechanisms, with evidence that sodium-glucose cotransporter 2 (SGLT2) inhibition can improve endothelial cell bioenergetics and reduce mitochondrial oxidative stress in mechanistic studies. This supports a pragmatic pathway in which clinically validated drugs may deliver partial “mitochondrial rejuvenation” alongside improvements in clinical outcomes^[94]^. Collectively, these approaches align with the mechanistic therapeutic objectives illustrated in Figure 4, targeting mitochondrial structure, reserve capacity, metabolic coupling, and stress tolerance.

### Mitochondrial-targeted clinical trials: lessons from past antioxidant failures

Early clinical efforts to target mitochondrial dysfunction in cardiovascular disease relied largely on systemic antioxidant supplementation, including vitamins C and E or beta-carotene. Despite strong mechanistic rationale and promising preclinical findings, large randomized trials generally failed to demonstrate cardiovascular benefit. One major reason for this failure is now understood to be the lack of mitochondrial specificity. Conventional antioxidants distribute widely throughout the cell and circulation and do not effectively accumulate within mitochondria, where the majority of ROS involved in cardiovascular aging are generated. Moreover, indiscriminate suppression of ROS may disrupt physiologic redox signaling that is necessary for adaptive stress responses, endothelial signaling, and metabolic regulation^[95,96]^.

In contrast, newer therapeutic strategies specifically target mitochondrial biology and therefore address the underlying mechanisms of mitochondrial network dysfunction. Mitochondria-targeted antioxidants such as MitoQ are designed to accumulate within the mitochondrial matrix via membrane-potential-dependent uptake, allowing more direct modulation of mitochondrial redox balance^[97]^. Similarly, the cardiolipin-interacting peptide elamipretide (SS-31) stabilizes IMM architecture and improves ETC efficiency, while interventions such as urolithin A enhance mitochondrial turnover by stimulating mitophagy pathways^[9,98]^. Early human trials of these mitochondria-targeted strategies have demonstrated improvements in vascular function, mitochondrial biomarkers, and functional performance in aging populations. These findings suggest that therapies that restore mitochondrial quality control and bioenergetic reserve, rather than broadly suppressing oxidative stress, may provide a more effective translational pathway for improving cardiovascular resilience during aging.

## UNDERSTANDING THE NETWORK IN MITOCHONDRIA: KEYS TO YOUTHFUL ORGANELLES

### Therapeutic objective: preserving mitochondrial reserve to sustain cardiac resilience

Youthful organelles can be operationally defined in cardiovascular terms by their capacity to sustain energetic flexibility, stress-responsive reserve, and quality-control competence rather than by maximal basal ATP production alone. The essential therapeutic goal is not to maximize basal ATP in resting myocardium, but to preserve the reserve capacity and stability margins that allow mitochondria to meet abrupt, beat-to-beat demand without triggering maladaptive signaling or injury. In the aging heart, vulnerability emerges when the energetic system approaches critical thresholds^[99]^, including loss of spare respiratory capacity^[100]^, increased electron leak and lipid peroxidation^[101]^, impaired Ca^2+^-metabolic coupling^[100]^, and a lowered barrier to permeability transition such that physiologic stresses (adrenergic drive, transient ischemia, pressure overload) precipitate disproportionate dysfunction^[102]^. Accordingly, a “young” mitochondrial state can be defined as one that maintains (i) high-flux oxidative phosphorylation with low leak^[103]^, (ii) preserved inner-membrane architecture and lipid quality^[104]^, (iii) competent quality-control turnover^[46]^, and (iv) a wide operating window between adaptive Ca^2+^-stimulated metabolism, transient permeability, [Figure 4] and catastrophic mitochondrial permeability transition pore (mPTP) opening^[105]^. These intervention priorities span preservation of inner-membrane architecture, cardiolipin stabilization, enhancement of spare respiratory capacity, optimization of Ca^2+^-metabolic coupling, and elevation of the mPTP opening threshold [Figure 4].

### Inner-membrane architecture as a determinant of high-flux efficiency

Youthful mitochondria support high-velocity energy transfer because their inner membranes maintain sharp cristae curvature and cardiolipin (CL)-rich microdomains that pack the ETC and ATP synthase into kinetically favorable assemblies. Rows of ATP synthase dimers sculpt the cristae ridge, locally retaining protons and shortening the diffusion path between proton pumps and F1Fo-ATP synthase^[106,107]^; OPA1 and mitochondrial contact site and cristae organizing system (MICOS) help preserve this architecture, which in turn stabilizes ATP synthase oligomers under load^[108]^. When this mesoscale order is intact, electron flux can be rapidly increased without excessive leak^[109]^. CL is central at each step: it binds complexes I, III, and IV and promotes their higher-order association into respiratory supercomplexes, reducing intercomplex diffusion barriers and minimizing electron slip and ROS^[110]^. With aging and oxidative stress, CL becomes peroxidized and partially depleted, cristae flatten, and supercomplexes destabilize. As a result, complex activities and carrier proteins [e.g., adenine nucleotide translocase (ANT)] lose CL-dependent support, raising resistance to proton pumping and lowering the ceiling of oxidative phosphorylation (OXPHOS) throughput even when basal ATP is maintained^[111]^. Experimental restoration of CL content or biophysical properties, or reinforcement of cristae curvature, correspondingly improves coupling efficiency and peak respiratory flux^[9]^.

### Reserve capacity and Ca^2+^-coupled metabolism define the stress-tolerance window

The earliest functional signature of this architectural erosion is loss of reserve (spare) respiratory capacity, the difference between maximal and basal oxygen consumption, rather than a fall in resting ATP. Reserve capacity integrates multiple control points spanning substrate delivery, NADH/reduced flavin adenine dinucleotide (FADH_2_) supply, electron transfer kinetics, proton pumping, and ATP synthase conductance; aging narrows this margin, so mitochondria appear competent at baseline yet fail during stress tests (adrenergic stimulation, ischemia-reperfusion, or uncoupler-driven demands for maximal flux)^[99,112]^. In cardiomyocytes, stress tolerance also depends on tight coupling of cytosolic Ca^2+^ signals to mitochondrial metabolism while remaining safely below the threshold for permeability transition^[113]^. Matrix Ca^2+^ uptake through the mitochondrial calcium uniporter (MCU) activates TCA-cycle dehydrogenases (including pyruvate dehydrogenase via pyruvate dehydrogenase phosphatase 1 (PDP1), NAD-isocitrate dehydrogenase, and 2-oxoglutarate dehydrogenase), thereby matching reducing-equivalent supply to contractile work on short timescales; impairment of this axis blunts workload-induced ATP augmentation^[113]^. With age, redox tone and microdomain organization shift in ways that allow Ca^2+^-driven stimulation to more readily co-activate ROS production and lower the threshold for mPTP opening, increasing susceptibility to ΔΨ collapse and injury during stress, even when resting energetics appear preserved^[114]^. Preserving CL integrity and cristae architecture, therefore, expands the operating window between adaptive Ca^2+^-stimulated flux and catastrophic permeability transition^[108]^.

## MITOCHONDRIA AS SENTINELS IN CARDIOVASCULAR AGING

Mitochondria act as sentinels in cardiovascular aging because they sit at the intersection of energy supply, redox control, and inflammatory triggering - three constraints that determine whether stress is resolved cleanly or converted into chronic dysfunction. In the heart, mitochondrial performance sets the ceiling for contractile reserve during adrenergic drive, pressure overload, or transient ischemia^[115]^. In the vasculature, mitochondrial redox signaling helps determine NO bioavailability and the endothelial inflammatory tone that governs perfusion and vascular homeostasis^[32]^. When these mitochondrial functions are preserved, the system retains a wide safety margin: routine fluctuations in workload are buffered without durable shifts in gene programs or tissue structure. With aging, that margin narrows^[29]^. The same stressors increasingly produce disproportionate oxidant signaling, impaired vasodilatory responses, and activation of sterile inflammatory pathways that then reinforce metabolic and vascular dysfunction^[33]^.

**Figure 5.**
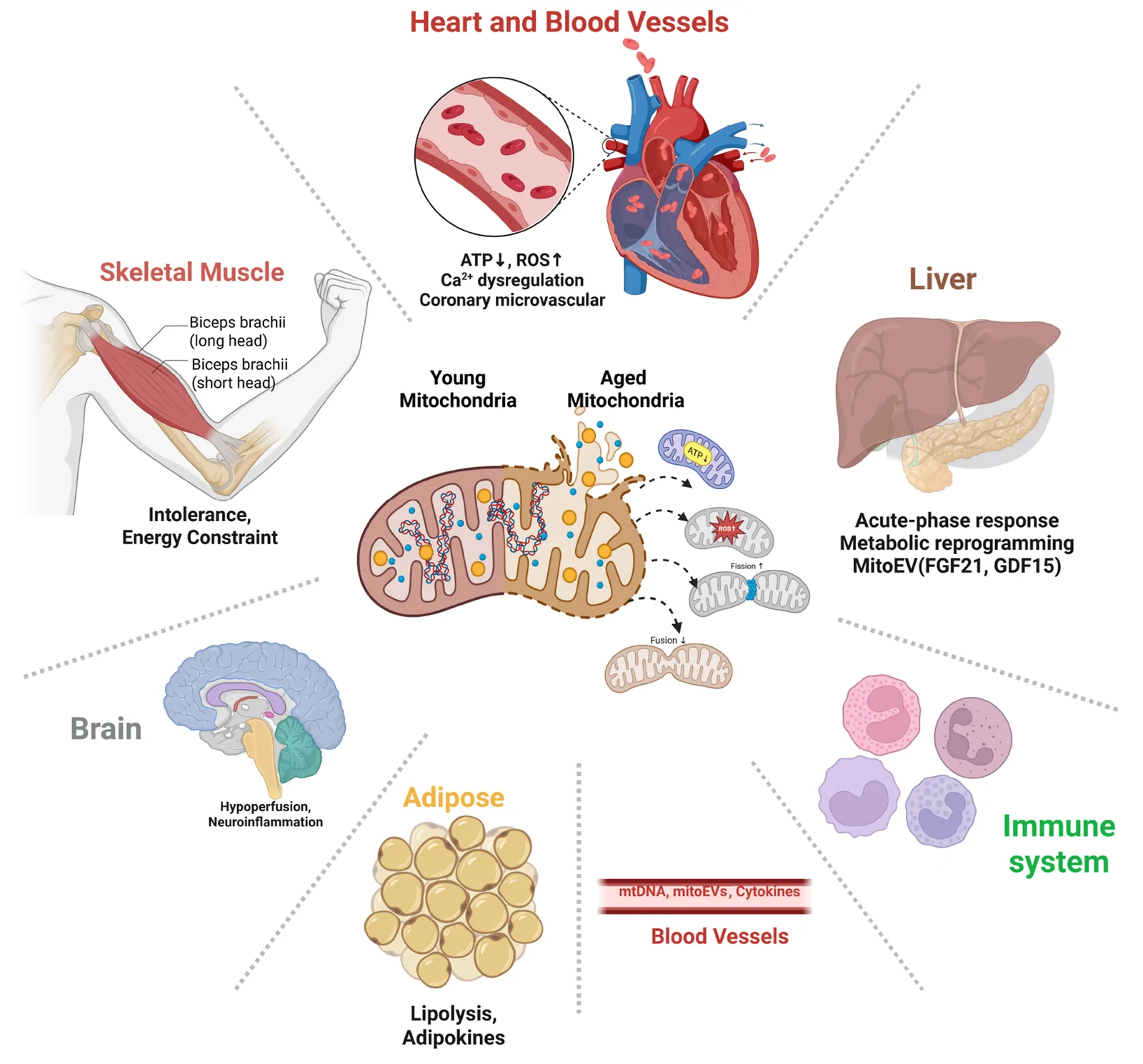
Organ-level consequences of mitochondrial aging and inter-organ communication networks. (Created in BioRender. Ahmad D (2026) https://BioRender.com/he5mt77). ATP: Adenosine triphosphate; ROS: reactive oxygen species; mitoEVs: mitochondrial extracellular vesicles; mtDNA: mitochondrial DNA; FGF21: fibroblast growth factor 21; GDF15: growth differentiation factor 15.

Across cell types, the most influential mitochondrial network effects for cardiovascular aging are often best framed through the microvascular endothelium, including coronary microvascular endothelial cells, with consequences that extend across heart, skeletal muscle, brain, liver, adipose tissue, and immune compartments [Figure 5]. These cells regulate local blood flow distribution, barrier properties, leukocyte trafficking, and paracrine signaling that shape the tissue environment in which cardiomyocytes must operate^[116]^. Endothelial mitochondria do not primarily exist to sustain bulk ATP demand. Instead, they are potent redox and signaling nodes^[32]^. A recurring theme in vascular aging is that greater reliance on mitochondrial-derived oxidant signaling accompanies reduced NO-dependent, vasoprotective signaling, contributing to endothelial dysfunction and impaired microvascular control^[33]^. In practical terms, once microvascular endothelial signaling drifts toward higher oxidant tone and lower NO availability, the heart becomes more vulnerable even if cardiomyocyte energetics are only modestly impaired, because oxygen and substrate delivery become less well matched to demand^[116]^.

Cardiomyocytes remain essential to the story because their mitochondria supply the rapid, high-flux energy conversion required for beat-to-beat work. Aging-associated changes in mitochondrial structure and turnover, especially when they slow the removal of damaged organelles and the replacement of lost capacity, push cardiomyocytes closer to a threshold at which otherwise tolerable stresses provoke larger drops in energetic performance and larger redox perturbations^[117]^. Reviews of vascular and cardiac aging repeatedly emphasize that mitochondrial dynamics and mitophagy shape whether stress leaves behind a persistent population of dysfunctional mitochondria that continues to generate maladaptive signals rather than allowing the system to return to baseline after the inciting event^[118]^.

A key mechanism linking mitochondrial injury to chronic cardiovascular inflammation is mislocalized mtDNA. When mitochondrial membranes or nucleoids are compromised, mtDNA can appear in the cytosol and engage innate immune DNA-sensing pathways such as cGAS-STING, which drives inflammatory transcriptional programs implicated across multiple cardiovascular disease settings^[119]^. This provides a direct route by which mitochondrial damage can shift both endothelial cells and cardiomyocytes toward a self-sustaining inflammatory state without infection, especially when aging-related declines in lysosomal clearance allow damaged mitochondrial material to persist^[120]^.

These mechanisms have led to the emerging concept of mitoinflammation, which refers to sterile inflammation driven by mitochondrial damage and the release of mitochondrial danger-associated molecular patterns. Because mitochondria retain bacterial evolutionary characteristics, including circular DNA enriched in unmethylated CpG motifs and cardiolipin-containing membranes, mitochondrial components released during cellular stress are readily recognized by innate immune sensors^[121,122]^. Cytosolic mtDNA can activate the cGAS-STING signaling, which triggers interferon responses and NF-κB-dependent inflammatory transcription. In parallel, mitochondrial damage can promote activation of the Nucleotide-binding oligomerization domain-like receptor family pyrin domain-containing 3 (NLRP3) inflammasome through mechanisms that involve mitochondrial ROS generation, potassium efflux, and oxidized mtDNA signaling^[123]^. In the aging heart, persistent mitochondrial dysfunction therefore converts metabolic stress into chronic inflammatory signaling and reinforces the systemic low-grade inflammatory state known as inflammaging. This mitochondrial immune interface provides a mechanistic link between declining bioenergetic function and age-associated cardiovascular remodeling, including endothelial dysfunction, fibrosis, and microvascular inflammation^[119]^.

When mitochondrial stress is prolonged, the response also extends beyond the local tissue through circulating stress-associated factors, often discussed as mitokines^[124]^. FGF21 and GDF15 are repeatedly described as factors induced downstream of mitochondrial dysfunction and integrated stress response signaling and can coordinate organism-level adaptations in metabolism and energy balance^[80]^. Sustained elevation is therefore better interpreted as a marker of unresolved mitochondrial strain than as a purely beneficial compensatory signal.

## ENDOTHELIAL AND MICROVASCULAR MITOCHONDRIA AS GATEKEEPERS OF CARDIAC RESILIENCE

**Figure 6.**
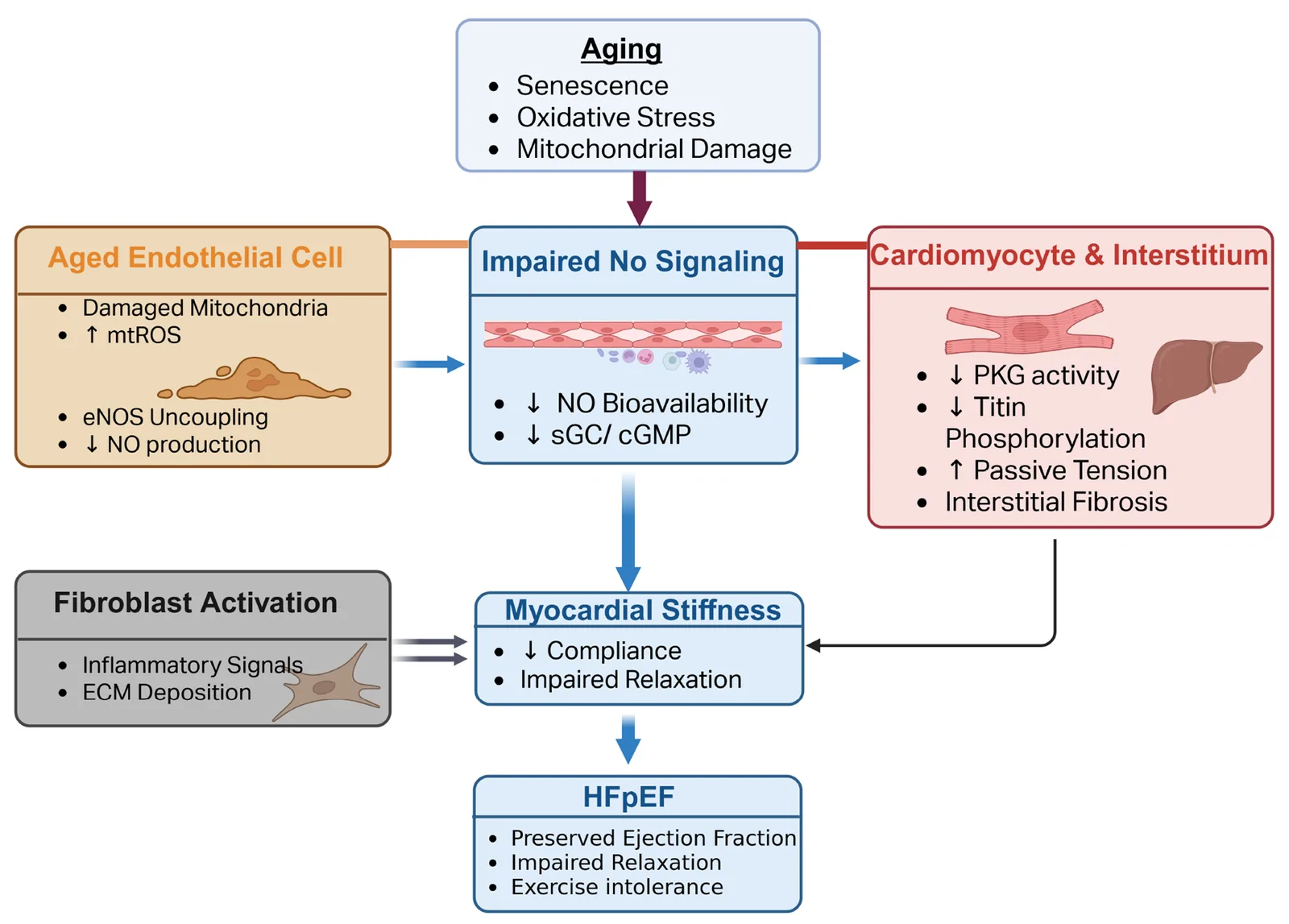
Endothelial to Myocardial Signaling in Cardiovascular Aging: Mitochondrial ROS, NO Deficiency, and Myocardial Stiffness Driving HFpEF (Created in BioRender. Shila TA (2026) https://BioRender.com/zlp092e). HFpEF: Heart failure with preserved ejection fraction; ROS: reactive oxygen species; eNOS: endothelial nitric oxide synthase; sGC: soluble guanylate cyclase; cGMP: cyclic guanosine monophosphate; PKG: protein kinase G; mtROS: mitochondrial reactive oxygen species; NO: nitric oxide; ECM: extracellular matrix.

Cardiac performance depends on tight coupling between perfusion and energy use, and converging evidence places the earliest failure in the coronary microcirculation, where endothelial and perivascular mitochondria set tone, flow reserve, and nutrient delivery. In HFpEF, impaired coronary flow reserve and reduced myocardial perfusion reserve identify a clinically meaningful phenotype even when epicardial arteries are unobstructed, consistent with a small-vessel-first pathogenesis that precedes chamber failure and amplifies myocardial energetic stress^[125]^. Endothelial mitochondrial dysfunction contributes by reducing oxidative phosphorylation flexibility and increasing ROS, which in turn promotes endothelial nitric oxide synthase (eNOS) uncoupling, loss of NO bioavailability, and vasomotor stiffness. Aging and cardiometabolic risk further deepen this redox shift, increasing superoxide and peroxynitrite that oxidize tetrahydrobiopterin and critical eNOS thiols, thereby converting eNOS from a NO source to a superoxide source^[126]^. In parallel, the endothelial glycocalyx thins and sheds under inflammatory and hemodynamic stress, blunting shear sensing and flow-mediated dilation and increasing leukocyte adhesion and albumin leak^[127]^. Multi-cohort and mechanistic studies in HFpEF show higher circulating markers of glycocalyx degradation, such as perlecan fragments^[128]^, and link endothelial injury to reduced coronary flow reserve and worse outcomes^[129]^. In parallel, endothelial mitochondrial ROS-driven NO deficiency has direct downstream consequences for the myocardium. Loss of endothelial NO bioavailability suppresses soluble guanylate cyclase/cyclic guanosine monophosphate/protein kinase G (sGC/cGMP/PKG) signaling in adjacent cardiomyocytes, favoring titin hypophosphorylation, increased passive tension, and impaired diastolic compliance, while chronic endothelial inflammatory activation promotes fibroblast activation and interstitial matrix deposition. Through this endothelial-to-myocardial signaling axis, microvascular mitochondrial dysfunction can be translated into myocardial stiffness and impaired relaxation, two central features of HFpEF [Figure 6].

Pericytes provide a second control layer that becomes critical as endothelial mitochondria age. These mural cells stabilize capillaries^[130]^, shape barrier properties^[131]^, and regulate capillary diameter through a contractile apparatus that responds to metabolic and redox cues^[132]^, thereby tuning local resistance and flow heterogeneity across the capillary network. Comparative work in brain and heart shows that pericyte-mediated capillary constriction can limit downstream perfusion after ischemia and likely in other pathological states, while experimental loss or phenotypic drift of cardiac pericytes initiates microvascular dysfunction, heightens endothelial inflammation, and contributes to diastolic dysfunction^[133]^. High-resolution studies and reviews demonstrate that pericytes influence angiogenesis, basement membrane composition, and capillary recruitment, and that their depletion or hypercontractility increases flow heterogeneity, lengthens oxygen diffusion distances, and reduces reserve during stress despite normal upstream conductance^[134]^. In clinical and preclinical contexts, these microcirculatory changes increase ischemia susceptibility and erode energetic reserve, providing a mechanistic bridge between capillary level dynamics and the exercise intolerance and demand ischemia that characterize HFpEF.

From a forward-looking perspective, endothelial aging should be conceptualized as a primary disease-modifying process that determines whether systemic cardiometabolic stress is buffered within the microcirculation or propagated to the myocardium as chronic energetic strain. A major priority for the field is mechanistic stratification of endothelial dysfunction into definable mitochondrial-linked failure modes^[135]^, including loss of mitochondrial redox control with eNOS uncoupling^[126]^, impaired mitochondrial turnover and stress tolerance^[136]^, disruption of NAD^+^-dependent signaling programs^[137]^, and mtDNA-associated innate immune activation, each of which is predicted to produce distinct signatures in flow reserve^[138]^, barrier permeability, and inflammatory activation^[139]^. In parallel, improved clinical phenotyping is needed to connect microvascular pathobiology to outcomes using integrated measures of coronary flow reserve and perfusion reserve alongside circulating or imaging-based indices of endothelial injury, including glycocalyx degradation and mitochondrial stress biomarkers^[129]^. This framework motivates a translational agenda for the next section in which candidate therapies are evaluated not only by hemodynamic endpoints, but also by their capacity to restore endothelial mitochondrial homeostasis, preserve glycocalyx mechanotransduction, and normalize pericyte-dependent capillary recruitment, thereby re-establishing perfusion-metabolism coupling before irreversible myocardial remodeling and HFpEF progression occur.

## HUMAN EVIDENCE AND MEASUREMENT: DEFINING MITOCHONDRIAL AGE

Defining “mitochondrial age” in humans is most informative when anchored in functional reserve and stress responses rather than static measures of mitochondrial content. At the tissue level, dynamic phosphorus magnetic resonance spectroscopy (MRS) can quantify post-exercise phosphocreatine recovery kinetics, which serve as an *in vivo* index of oxidative phosphorylation capacity because phosphocreatine resynthesis is driven by mitochondrial ATP production; slower recovery reflects reduced oxidative capacity and a more aged bioenergetic phenotype even when resting ATP appears preserved^[140]^.

At the cellular scale, optical metabolic imaging approaches such as fluorescence lifetime imaging of endogenous NAD(P)H (reduced nicotinamide adenine dinucleotide phosphate and reduced nicotinamide adenine dinucleotide) and flavin adenine dinucleotide (FAD) can report enzyme engagement and redox state by distinguishing bound and free lifetime components and by tracking redox ratio shifts across cells and tissues, enabling label-free detection of metabolic state changes in translational samples^[141]^. In blood, peripheral immune-cell bioenergetic profiling offers an accessible “systemic stress test” of mitochondrial resilience, including reserve and spare respiratory capacity measured by respirometric methods; this approach is increasingly used to relate mitochondrial function to aging phenotypes, frailty, and cardiometabolic risk^[142]^.

Circulating cell-free mtDNA provides an additional minimally invasive window into mitochondrial damage signaling and sterile inflammation in cardiovascular contexts, but it requires careful interpretation because levels are sensitive to preanalytical handling and to the specific physiological or disease context in which mitochondrial material is released^[143]^. Human studies also emphasize key modifiers that must be incorporated into trial design to improve signal-to-noise and interpretability, including tissue specificity of mitochondrial aging, sex-related differences in mitochondrial bioenergetics, and circadian regulation of metabolism and vascular function^[144–146]^.

For clinical translation, measurement should follow a mechanism-anchored playbook. Endpoints should be paired to the hypothesized therapeutic lever and captured under standardized conditions, with core-laboratory adjudication when feasible. For endothelial coupling, brachial artery flow-mediated dilation remains a widely used, shear-dependent, nitric-oxide-linked measure with established expert consensus recommendations that improve reliability when protocols are standardized^[147]^. For mitochondrial functional reserve, practical trial panels can combine (i) phosphocreatine recovery kinetics from 31P MRS as a tissue oxidative-capacity readout, (ii) peripheral blood cell spare respiratory capacity as a systemic resilience metric, (iii) NAD(P)H and FAD lifetime metrics for cellular redox and enzyme engagement, and (iv) circulating cell-free mtDNA as a damage and inflammatory-signal readout, with all components normalized to age and sex and measured at consistent circadian time points^[140,143,147–149]^. An optional vascular-coupling subscore can integrate endothelial function with perfusion or coronary flow reserve, where microvascular supply-demand matching is central.

Patient enrichment should similarly be mechanism-driven. Early phenotypes in which mitochondrial and microvascular lesions dominate, such as HFpEF with exercise-limited hemodynamics or microvascular angina with impaired perfusion reserve despite unobstructed epicardial arteries, are plausible settings in which mitochondria-targeted therapies may yield clearer functional signals than in advanced remodeling states.

## NEW PERSPECTIVES ON IMPROVING MITOCHONDRIAL YOUTHFULNESS AND THE MEASUREMENT PRECISION

A coherent picture has begun to emerge in which mitochondrial quality control is a modifiable driver of clinical aging, and the features of a minimum effective rejuvenation strategy can be inferred from human and translational data. Restoration of inner-membrane quality that supports cristae curvature and cardiolipin binding appears central, because cardiolipin stabilizes respiratory supercomplexes and improves electron transfer efficiency^[9,150]^. In failing human hearts, the cardiolipin-interacting tetrapeptide elamipretide improved complex I and IV activities, supercomplex-linked respiration, and overall oxygen flux in *ex vivo* assays of biopsy tissue^[9]^, indicating that relatively small biophysical shifts at the membrane can translate into measurable gains in flux capacity that matter under stress. Parallel evidence supports the mitophagy arm of quality control. In randomized trials in middle-aged and older adults, supplementation with urolithin A increased performance and endurance while improving biomarkers of mitochondrial health that reflect enhanced mitophagy, establishing target engagement and functional benefit in humans without disease-stage confounders^[90]^. At the failure end of the spectrum, age-dependent sensitization of the mPTP lowers the threshold for stress-induced loss of membrane potential in the heart, linking impaired quality control to vulnerability during ischemia-reperfusion or adrenergic load. Raising this threshold is therefore a plausible criterion for effective rejuvenation and is more closely linked to functional outcomes than static content measures^[151]^. These studies suggest that a minimally effective package will likely include membrane-level remediation that restores cardiolipin-supported architecture, measurable increases in mitophagy with preserved lysosomal clearance, and a demonstrable upward shift in the permeability transition threshold, with clinical translation judged by gains in stress-tested reserve rather than resting surrogates. A summary of mitochondria-targeted therapeutic strategies, their mechanistic targets, and current clinical status is provided in Table 2.

Coordination across the myocardial syncytium has been most clearly described at the endothelial-cardiomyocyte interface, and the literature points to shared signals that can be mapped and perturbed. Endothelial mitochondria help maintain NO bioavailability; with aging, redox shifts, and tetrahydrobiopterin oxidation promote eNOS synthase uncoupling, thereby reducing flow-mediated dilation and limiting perfusion reserve during stress^[152]^. Communication is not confined to classical mediators. Mitochondrial content and signals also traffic between cell types in EVs that can carry mtDNA and proteins. This vesicle export increases when lysosomal clearance is constrained, providing a route by which local quality-control bottlenecks propagate inflammatory tone and metabolic cues across the vessel wall and into the interstitium^[153]^. Systemic coordination is further suggested by mitochondrial stress-induced secreted factors, including the mitokines FGF21 and GDF15, which are transcriptionally induced by integrated stress responses and secreted by stressed tissues to couple organelle dysfunction to whole-body metabolic adaptation^[80]^. By contrast, mitochondria are not considered a canonical secretory source of cytokines; mitochondrial damage more commonly amplifies cytokine production indirectly through the release of mitochondrial danger-associated molecular patterns (DAMPs), such as mtDNA, that engage innate immune sensors^[119]^. Mitokines are mitochondrial stress-induced secreted factors, and the term is functional rather than taxonomic; it overlaps with, but is not synonymous with, cytokines, as shown by GDF15, a stress-induced cytokine of the transforming growth factor-beta (TGF-β) superfamily, and FGF21, an endocrine metabolic hormone, both of which rise in response to mitochondrial dysfunction^[124]^. Mitochondria are not generally thought to secrete cytokines directly; instead, mitochondrial stress and damage promote cytokine production by exporting mitochondrial danger signals such as mtDNA that activate innate immune pathways including cGAS-STING and the NLRP3 inflammasome^[154]^. A working model therefore envisions endothelial mitochondria as gatekeepers of shear sensing and NO signaling that communicate with cardiomyocytes through redox and calcium microdomains, through metabolite exchange such as lactate and ketone use, and through vesicle traffic and secreted-factor signaling, including cytokines and mitokines, which together reflect sustained stress when local repair falls behind.

Measurements of mitochondrial aging should be investigated using standardized, mechanism-anchored frameworks. Progress will depend on reporting standards that allow mitochondrial endpoints to be compared and pooled across cardiovascular trials. For energy, 31P MRS of phosphocreatine recovery after brief exercise is reproducible, correlates with in vitro oxidative capacity, and should be paired with standardized measures of oxygen consumption (VO_2_) kinetics during ramp exercise and with myocardial perfusion or coronary flow reserve obtained under defined shear or adrenergic stimuli. For endothelial function, consensus statements on flow-mediated dilation and related measures provide procedures, calibration steps, and diagnostic thresholds that can be harmonized across sites; adherence to these guidance documents reduces variability and improves interpretability of shear-dependent endpoints directly tied to endothelial mitochondrial state through NO biology. For quality, trial reports should include validated proxies of cardiolipin status when tissue is available, high-resolution respirometry signatures that separate leak, coupling, and spare capacity, and dynamic markers of mitophagy and lysosomal flux. Interpretation should also account for the possibility that release of mitochondrial material in large EVs reflects downstream clearance constraints. For signaling, preanalytical handling and timing for circulating cell-free mtDNA and redox and acetylation panels should be specified, and endothelial outcomes should be adjudicated by a core laboratory to permit cross-trial synthesis. With these standards, the field will be better positioned to quantify the smallest effective quality-control rejuvenation that improves patient function and to test coordinated interventions across endothelial and myocardial compartments.

## CONCLUSION

Cardiovascular aging can be understood as a progressive failure of a mitochondrial network that spans the myocardium, endothelium, and microvasculature. Across the literature reviewed here, three linked domains consistently account for this loss of resilience. Energy declines when spare respiratory capacity contracts and perfusion-metabolism coupling become unreliable under load, even while resting ATP remains near normal. Quality falters when cristae and cardiolipin architecture deteriorate, when fission-fusion balance drifts, and when mitophagy and lysosomal clearance cannot keep pace with damage. Signaling becomes maladaptive when mtDNA and peptides trigger innate immunity through cGAS-STING, when compartment-specific NAD^+^-SIRT control slips, and when stress-induced cytokines and mitokines chronically reinforce cellular stress programs. These processes interact most visibly in endothelial and microvascular territories that gate flow and barrier function, thereby setting an early ceiling on oxygen delivery that is then inherited by cardiomyocytes during exercise or ischemia. Viewing the problem through this network lens replaces a single-lesion narrative with a framework of tractable nodes and links that can be measured, perturbed, and followed over time. Translation should proceed by measuring what matters, intervening where the earliest failures occur, and defining success by restored function under stress. The proposed Mitochondrial Functional Age panel integrates dynamic energy metrics, markers of organelle quality and turnover, and indices of signaling tone, while incorporating circadian timing and site standards to support comparability. Such a composite can enrich trials for early phenotypes characterized by perfusion-metabolism uncoupling, and it can anchor go-no-go decisions to mechanisms rather than to distant outcomes alone. Interventions that increase reserve capacity, stabilize cristae and cardiolipin architecture, and normalize mitophagy-lysosome flux, together with endothelial-targeted strategies that recover NO biology and glycocalyx integrity, are most likely to improve clinically relevant endpoints such as exercise hemodynamics, diastolic reserve, and recovery kinetics. Genetic and nucleic acid-based approaches remain promising but will require careful attention to delivery, safety, and dose control. If these elements are adopted, mitochondrial youthfulness can become a practical therapeutic objective rather than a merely descriptive label. A field that routinely quantifies reserve, quality control, and signaling, targets endothelial and intermyofibrillar microdomains, and judges success by improved coupling of perfusion to work will be positioned to convert mechanism into medicine. The expected payoff is not only the delayed onset of overt disease but also measurable gains in everyday cardiovascular function in older adults.

## Figures and Tables

**Table 1.** Integration of mitochondrial quality control pathways in cardiovascular aging

System	Key components	Function	Failure mechanism in aging	Downstream consequence
NAD^+^/Sirtuin signaling	NAD^+^, SIRT1, SIRT3	Regulates mitochondrial metabolism, antioxidant defense, and transcription of mitochondrial genes	Age-dependent NAD^+^ decline reduces sirtuin activity	Impaired mitochondrial biogenesis and reduced mitophagy signaling
AMPK energy sensing	AMPK, PGC-1α	Detects energetic stress and stimulates mitochondrial biogenesis and autophagy	Reduced AMPK responsiveness in aging	Decreased mitochondrial turnover and metabolic flexibility
Mitochondrial dynamics	DRP1, MFN1/2, OPA1	Controls mitochondrial fission and fusion to segregate damaged organelles	Dysregulated dynamics promote fragmentation or hyperfusion	Impaired isolation of damaged mitochondria
Mitophagy pathways	PINK1/Parkin, BNIP3/NIX, FUNDC1	Selective removal of dysfunctional mitochondria	Reduced mitophagy signaling and lysosomal flux	Accumulation of dysfunctional mitochondria
Lysosomal degradation	Autophagosome–lysosome pathway	Final degradation step for mitochondrial clearance	Lysosomal decline with aging	Incomplete mitochondrial turnover and inflammatory signaling

NAD^+^: Nicotinamide adenine dinucleotide; SIRT1: sirtuin 1; SIRT3: sirtuin 3; AMPK: adenosine monophosphate-activated protein kinase; PGC-1α: peroxisome proliferator-activated receptor gamma coactivator 1-alpha; DRP1: dynamin-related protein 1; MFN1/2: mitofusin 1/2; OPA1: optic atrophy protein 1; PINK1: PTEN-induced kinase 1; Parkin: E3 ubiquitin-protein ligase Parkin; BNIP3: BCL2/adenovirus E1B 19-kDa-interacting protein 3; NIX: BNIP3-like protein; FUNDC1: FUN14 domain-containing protein 1.

**Table 2.** Mitochondria-targeted therapeutic strategies in cardiovascular aging

Intervention	Mechanistic target	Primary mito lever (energy/quality/signaling)	Model/study type	Key cardiovascular findings	Clinical status
MitoQ (mitoquinone)^[155]^	Mitochondrial ROS modulation	Signaling/Energy	Randomized controlled trial in healthy older adults	Improved brachial artery flow-mediated dilation; reduced aortic stiffness	Completed human RCT
Elamipretide (SS-31)^[9]^	Cardiolipin stabilization; cristae integrity	Energy/Quality	*Ex vivo* failing human myocardium; Barth syndrome trials	Improved complex I/IV activity and supercomplex respiration; enhanced mitochondrial oxygen flux	FDA accelerated approval (Barth syndrome)
Urolithin A^[90,98]^	Mitophagy activation	Quality	Randomized placebo-controlled trials in middle-aged and older adults	Improved muscle endurance; increased biomarkers of mitochondrial health	Human RCT completed
Nicotinamide riboside (NR)^[91]^	NAD^+^ repletion; sirtuin activation	Energy/Signaling	Human pilot RCT in hypertensive middle-aged/older adults	Improved vascular parameters; enhanced metabolic resilience	Early-phase human trials
AMPK activators (exercise, pharmacologic)^[156]^	Energy sensing; mitochondrial biogenesis	Energy/Quality	Animal aging models; human exercise studies	Increased mitochondrial content and respiratory capacity; improved diastolic reserve	Established lifestyle therapy; pharmacologic agents under study
Time-restricted eating (TRE)^[92]^	Nutrient-sensing alignment; metabolic stress modulation	Energy/Signaling	Human interventional studies in older adults	Improved endothelial function; vascular coupling endpoints under evaluation	Ongoing clinical trials
SGLT2 inhibitors (e.g., empagliflozin)^[94]^	Improved mitochondrial redox state; endothelial bioenergetics	Energy/Signaling	Mechanistic human studies; diabetic mouse models; large CV outcome trials	Improved endothelial function; reduced oxidative stress; improved HF outcomes	FDA-approved for HF and diabetes
Mitophagy enhancers (e.g., BNIP3/NIX pathway targeting, experimental compounds)^[157,158]^	Selective mitochondrial clearance	Quality	Transgenic mouse models of impaired mitophagy	Restored mitochondrial turnover; improved cardiac stress tolerance	Preclinical stage
mPTP modulation strategies^[159]^	Increase permeability transition threshold	Quality/Energy	Aged rodent heart models	Reduced ischemia-reperfusion injury; improved stress resilience	Preclinical/experimental

MitoQ: Mitoquinone/mitochondria-targeted ubiquinone; ROS: reactive oxygen species; RCT: randomized controlled trial; SS-31: Szeto-Schiller peptide 31; FDA: food and drug administration; NR: nicotinamide riboside; TRE: time-restricted eating; SGLT2: sodium-glucose cotransporter 2; CV: cardiovascular; HF: heart failure; mPTP: mitochondrial permeability transition pore; complex I/IV: mitochondrial respiratory chain complexes I and IV.

## Data Availability

Not applicable
